# Laparoscopic Excision of Large Intra-Abdominal Cysts in Children: Needle Hitch Technique

**DOI:** 10.1155/2015/937191

**Published:** 2015-12-21

**Authors:** Brice Antao, Jeffrey Tan, Feargal Quinn

**Affiliations:** ^1^Department of Paediatric Surgery, Our Lady's Children's Hospital, Crumlin, Dublin 12, Ireland; ^2^Department of General Surgery, Waikato Hospital, Hamilton 3204, New Zealand

## Abstract

Laparoscopic surgery has both diagnostic and therapeutic advantages in the management of intra-abdominal cysts in children. Large cysts in small children pose technical challenges during laparoscopic surgery, requiring multiple incisions and advanced laparoscopic skills. This paper describes a novel laparoscopic technique using minimal manipulation for both aspiration and excision of the cyst. This simple, safe, and effective approach was used to achieve traction and facilitate excision of a large intra-abdominal cyst in a neonate and a young child.

## 1. Introduction

Giant intra-abdominal cysts in children are rare and most commonly arise from the small bowel mesentery, the omentum, or the ovary. Optimal surgical management requires complete excision of these lesions. Although they are invariably benign, a full laparotomy has been the conventional approach for resection, often via a large midline incision given the size of these cysts. The advent of minimally invasive surgery has allowed resection of these cysts, without need for a full laparotomy, with the benefit of improved cosmesis, less postoperative pain, and shorter hospital stay.

However, laparoscopy can be technically challenging in small children with large intra-abdominal cysts. This is mainly due to lack of intra-abdominal space and poor ergonomics in relation to port placements in smaller children with large cysts. We describe a needle hitch technique, which facilitated excision of large intra-abdominal cysts in both a neonate and a young child.

## 2. Patients and Methods

Two female patients (4 weeks old, 4 years old) underwent laparoscopic excision of large intra-abdominal cysts using a needle hitch technique. The case reports of both these cases are outlined below.

### 2.1. Case  1

**Figure 1:**
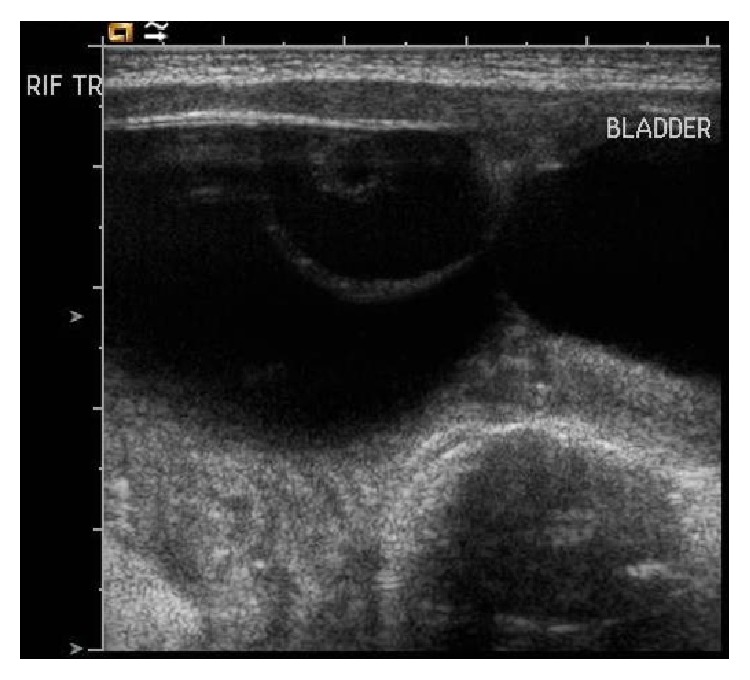
Abdomen and pelvic ultrasonography, showing a complex large cystic mass arising from right ovary approximately 7 cm in diameter. There is a 2 cm thin walled daughter cyst within the large cyst.

A premature female neonate born at 34-week gestation was referred with an antenatally detected abdominal cystic mass. She underwent postnatal abdominal and pelvic ultrasonography on day 2 of life, which demonstrated a cystic mass in the lower abdomen with a maximum diameter of 7 cm. The exact origin of this structure was uncertain. On clinical examination, her abdomen was distended, with a diffuse palpable mass occupying her entire abdomen. She continued to remain asymptomatic and underwent a repeat ultrasonography 3 weeks later, which suggested this cystic mass to be arising from the right ovary extending from the right side of the pelvis up to the inferior surface of the liver. There was no reduction in size compared to the previous ultrasonography. However, it now had a complex appearance of a small 2 cm daughter cyst with the larger cyst with some normal ovarian tissue. Also during ultrasonography examination, the position of the normal ovarian tissue kept changing from superior to inferior aspect of the mass with change of position, suggestive of a risk intermittent torsion (Figure 1). Her serum alpha-fetoprotein, beta-HCG, and LDH levels were within normal limits for her age. In view of this, she underwent a laparoscopic excision of right ovarian cyst with salvage of the rest of her ovary. The procedure took 40 minutes and there were no intraoperative or postoperative complications. She was discharged home after 2 days and has been followed up at regular intervals over the last year. The histology of the resected cyst confirmed it to be a simple follicular cyst of her ovary. Her most recent pelvic ultrasound scan demonstrates a normal ovary on both sides with no recurrence.

### 2.2. Case  2

**Figure 2:**
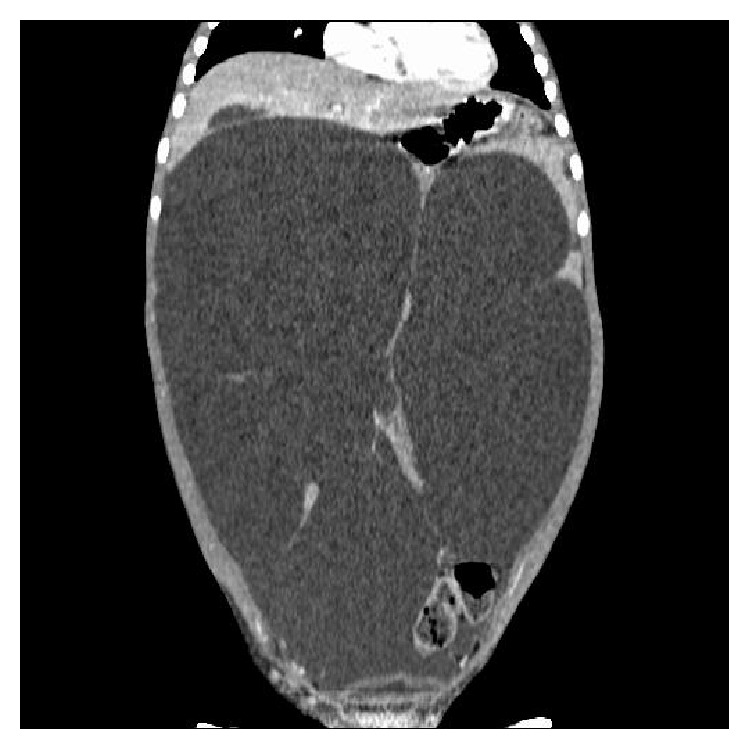
CT scan of abdomen and pelvis showing a giant multiloculated cystic mass occupying the entire abdomen with mass effect on the surrounding structures.

A 4-year-old girl was referred with a long-standing history of over a year of intermittent vomiting, abdominal pain, and progressive abdominal distension. She was born at term following an uneventful pregnancy and normal antenatal ultrasonography. On clinical examination, she had very marked abdominal distension and a visible and palpable diffuse mass occupying her entire abdomen. She was initially investigated with an abdominal and pelvic ultrasonography which demonstrated a large multilocular fluid filled cystic mass occupying most of her abdomen from the left upper quadrant to her pelvis and extending across the lateral aspect of her abdomen and lying anterior to her bladder. A CT scan of her abdomen and pelvis confirmed a 27 cm × 20 cm × 14 cm multilocular mass extending from the left upper quadrant to her left iliac fossa, with bowel loops invaginating in between, causing mass effect on the rest of the her abdominal contents including her IVC (Figure 2). She underwent a laparoscopic near total excision of her abdominal cyst. At laparoscopy, the cyst was seen to be enveloping the entire small bowel mesentery, in close relation to the bowel wall. In the portion of the cyst within the root of the mesentery, where it was difficult to achieve complete excision without compromising the vascularity of the bowel, a partial excision was done with electrocauterization of the residual mucosal lining. The operative time was 50 minutes, with no intraoperative complications or need for conversion to an open procedure. She made a good recovery following her surgery with no postoperative complications and was discharged home after 24 hours. Histological examination of the excised specimen revealed a cystic lymphangioma. At 6-month follow-up she continues to remain clinically well with no symptoms. A repeat abdominal ultrasonography suggested a small 2 cm recurrent cyst, which was well localized with the small bowel mesentery, with no mass effect. She continues to remain on regular follow-up with serial abdominal ultrasound scans.

## 3. Surgical Technique

**Figure 3:**
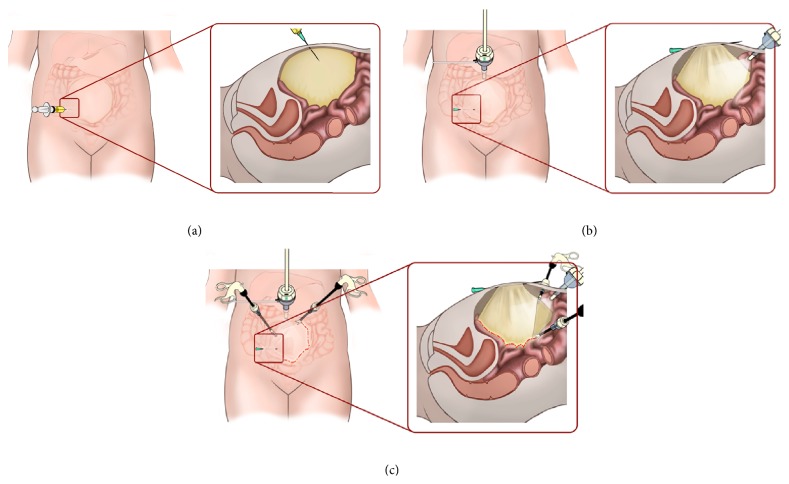
Diagrammatic depiction of the needle hitch technique. (a) Cyst aspirated via a direct percutaneous puncture using a spinal needle. (b) Under direct laparoscopic view, the needle is advanced through the anterior surface of the cyst wall and secured at the exit site through the skin with an artery clip. (c) The hitching technique maintains traction on the cyst wall and facilitates its dissection using instruments through the two working ports.

With the patient under general anaesthesia, a nasogastric tube and Foley's catheter were inserted. The patient was placed supine and in a reverse Trendelenburg position. Using an 18 Ga. × 3.5 in. (90 mm) spinal needle (SPINOCAN, B. Braun Medical, Bethlehem, Pennsylvania) a skin puncture was made from the right iliac fossa into the palpable cystic mass and the cyst was completely aspirated [case 1 (60 mLs), case 2 (3500 mLs)] (Figure 3(a)). This was done under ultrasound guidance in case 1. With the needle left in situ, a 5 mm umbilical port for the camera was inserted using an open Hasson technique. Following insufflation with CO_2_ to a pressure of 10 mm [case 1 (8 mm)], a 5 mm 30-degree laparoscope was introduced. Two further 5 mm working ports were introduced in the right and left upper quadrants. Under laparoscopic guidance, the needle within the cyst cavity was then advanced through the anterior cyst wall and the tip was brought out of the abdomen a few inches away from the entry point. The end of the needle was secure, close to the exit site near the skin with a fine-tipped artery forceps to prevent it from slipping back into the peritoneal cavity (Figure 3(b)). This hitching manoeuver held the cyst under traction to facilitate dissection, using instruments introduced through the two working ports (Figure 3(c)). Using a combination of Maryland dissecting forceps, hook diathermy, and Ligasure vessel-sealing system (Covidien, Minneapolis, Minnesota), the cyst was completely dissected out with good haemostatic control. For case 2, given the extensive size of the cyst the needle was repositioned across the anterior cyst wall in a sequential manner to provide continuous traction as the dissection proceeded through the length of the cyst. This was done without the need for repositioning the needle from its original entry site. Also, in case 2, the mucosa of a small portion of residual cyst that was left in the root of the mesentery was sclerosed using electrocautery. After complete excision of the cyst, the hitching needle was removed and the specimen was placed in a 5 mm specimen retrieval bag (INZII Applied Medical, Rancho Santa Margarita, California) and was retrieved via the umbilical port. The port sites were closed with 4/0 VICRYL sutures (Ethicon, Johnson & Johnson, Somerville, New Jersey) and INDERMIL tissue glue (Vygon, Swindon, County Wiltshire) to the skin.

## 4. Discussion

Intra-abdominal cysts can be categorized based on their location in either the solid organs, retroperitoneum, mesentery, or omentum. Ovarian cysts are more frequent, compared to mesenteric or omental cysts. Mesenteric cysts can occur anywhere in the mesentery of the gastrointestinal tract from the duodenum to the rectum, and they may extend from the base of the mesentery into the retro peritoneum. They most commonly occur in the ileal mesentery of the small bowel [1]. Omental cysts are confined to the lesser or greater omentum. Neonatal ovarian cysts are thought to arise secondary to increased hormonal stimulation from exposure to maternal oestrogen and excessive release of placental chorionic gonadotropin. With advances in antenatal ultrasonography, the incidence of neonatal intra-abdominal cysts has increased [2]. They are often diagnosed antenatally or as an incidental finding during laparotomy for another condition or when they become symptomatic.

Symptoms in children vary from abdominal distension, abdominal pain, or a palpable mass to small bowel obstruction or an acute life-threatening intra-abdominal catastrophe such as intestinal volvulus or infarction, ovarian torsion, or peritonitis as a result of rupture of the cyst.

Although, with the widespread use of prenatal ultrasonography, most of these intra-abdominal cysts are being diagnosed in utero, prenatal management of fetal ovarian cysts remains controversial. In cases of mesenteric and omental cysts discovered antenatally, intervention during early infancy is indicated to prevent complications such as obstruction and intestinal volvulus. There is no consensus regarding the optimal management of ovarian cysts in infants. The dilemma in the management of antenatally diagnosed ovarian cysts is predicting which cysts undergo spontaneous regression and which might lead to ovarian torsion and inherent loss of the ovary or even a fatal outcome. Seventy-five percent of neonatal ovarian cysts will undergo spontaneous resolution by 6 months of age [3]. Although symptomatic cysts demand intervention, simple asymptomatic cysts less than 5 cm in diameter can be left alone but reassessed ultrasonographically. If simple cysts are larger than 5 cm in diameter the risk of torsion may be significant (25%), and intervention often is advocated [3, 4]. Complex ovarian cysts are deemed to have an echogenic appearance on ultrasound and may contain septa, debris fluid level, or clot. Some studies advocate early surgical intervention for ovarian cyst with complex characteristics due to the higher risk of complications like bleeding, rupture, or intestinal obstruction [5, 6]. However, other studies dispute this by suggesting a high incidence of spontaneous resolution of complex cysts without any associating complications [7, 8].

In our cases, the decision for surgical intervention in the first child with ovarian cyst was because of findings on serial pelvic ultrasonography of persistent increase in size more than 5 cm, complex appearances of cyst, and change in position of normal ovarian tissue suggestive of possible intermittent torsion. In the second child, indication for surgery was symptoms as a result of mass effect of the cyst causing intermittent intestinal obstruction. Other common indications for surgical intervention include intracystic haemorrhage or infection [9].

The goal of surgical therapy is complete excision of the cyst. Omental cysts can be removed without damage to the adjacent bowel [10, 11]. The preferred treatment of mesenteric cysts is enucleation; however, intestinal resection may be required in up to 50–60% of cases, in order to maintain the viability of the rest of the bowel [12, 13]. If enucleation or resection is not possible because of the size of the cyst or because of its location deep within the root of the mesentery, the third option is partial excision with marsupialization of the remaining cyst into the abdominal cavity. If marsupialization is performed, the cyst lining should be sclerosed with 10% glucose solution, electrocautery, or tincture of iodine to minimize recurrence. Partial excision alone with or without drainage is associated with a high recurrence rate [2]. In our second child, given the size and extent of the cyst, complete excision was not possible, without compromising the vascularity of the bowel. Hence, most of the cyst was completely excised, and the remaining cyst lining was sclerosed with electrocautery. In cases of ovarian cysts in children, emphasis should be on sparing functional ovary and the use of ovarian sparing procedures [14–17]. Simple cysts should be fenestrated, while complex or functional cysts should be excised with preservation of the remaining ovary. Although ultrasound guided aspiration has been used in management of large simple ovarian cysts, its outcome is not definitive. Careful ultrasonographic follow-up and repeated aspirations may be necessary, which increases the risk of bleeding and infection in the cyst. Also, the diagnosis of the origin of these cysts is not always clear on ultrasonography [18].

Laparoscopic surgery has increasingly been used in the diagnosis and management of intra-abdominal cysts in children. However, there are several challenges, with laparoscopic approach, especially in cases of large cyst in relation to the size of the patient. It is often difficult to gain access to the peritoneal cavity for port placement and one encounters difficulty in intracorporeal dissection and manipulation of the cyst because of limited space in smaller children and the need for multiple incisions and instrumentations. Also chemical peritonitis may result from leakage of benign cyst fluid into the peritoneal cavity [19]. In order to address these issues, several laparoscopic approaches and modifications have been described. These include either drainage of the cyst by ultrasound guided paracentesis or drainage during laparoscopy followed by excision or manipulation of the cyst or extracorporeal cystectomy [20–25]. For drainage and manipulation of the cyst, different techniques have been described using a planned trocar placement through the cyst, percutaneous gastrostomy introduction set, soft cup aspirator set, suprapubic catheter, extracorporeal drainage via a minilaparotomy, and aspiration and traction through the port to facilitate dissection [20–25]. The technique we describe provides a controlled means of aspirating the cyst and allows traction to the cyst wall to facilitate intracorporeal manipulation and dissection of the cyst. Also, one can readjust the needle along the anterior cyst wall to provide traction in a sequential manner in cases of large cysts as was done in case 2. The needle hitch technique minimizes the need for additional instrumentation and ports for traction and facilitates better ergonomics for intracorporeal manipulation and dissection of large cysts.

Cystic lymphangiomas as seen in case 2 are sometimes clinically difficult to differentiate from mesenteric and omental cysts [26]. Cystic lymphangiomas have an endothelial cell lining, foam cells, and a thin wall that contains lymphatic spaces, lymphoid tissue, and smooth muscle. Mesenteric cysts lack smooth muscle and lymphatic spaces, and the cells lining the cysts are cuboidal or columnar in nature. An omental cyst has the same histological characteristics as a mesenteric cyst but is confined to the greater and lesser omentum. Lymphangiomas are more diffuse and occur in the mesentery or retroperitoneum, and patients may present with them earlier in life than those with mesenteric or omental cysts [26]. Long-term follow-up with ultrasonography scan is important, especially where complete excision has not been achieved because of risk of recurrence [2]. Even though the child with cystic lymphangioma (case 2) developed a recurrence, it is small and well localized compared to the initial presentation without any mass effect. Given that the child continues to remain asymptomatic and its extremely small size (<2 cm) we plan to follow up with this child with serial pelvic ultrasonography. If the child does develop symptoms related to mass effect of this recurrent cyst or there is an increase in size on serial ultrasound scans, we would elect to resect this cyst, using the same surgical technique described here.

## 5. Conclusions

Laparoscopic surgery has the advantages of being both diagnostic and therapeutic in the management of intra-abdominal cysts in children. The needle hitch technique is a simple, safe, and effective approach in the management of large intra-abdominal cysts in children. This technique provides all the benefits of minimally invasive surgery including better cosmesis, less pain, and shorter hospital stay. It is an effective variation of previously described methods, requiring minimal manipulation for both aspiration and traction, limiting the need for multiple ports and instrumentations. This has the technical advantage of providing better ergonomics for the dissection and manipulation of large intra-abdominal cysts and facilitates the use of the laparoscopic approach in children of all ages, irrespective of the size of the cyst.
